# Mitochondrial bioenergetics and networks in melanoma: an update

**DOI:** 10.1007/s10495-025-02155-4

**Published:** 2025-07-28

**Authors:** Gaia Giannitti, Alyssa Julia Jennifer Paganoni, Sara Marchesi, Riccardo Garavaglia, Fabrizio Fontana

**Affiliations:** Department of Pharmacological and Biomolecular Sciences “Rodolfo Paoletti”, Università Degli Studi di Milano, Milan, Italy

**Keywords:** Melanoma, Mitochondrial metabolism, Mitochondrial dynamics, Redox homeostasis, Apoptosis, Cancer therapy

## Abstract

Melanoma is the most aggressive and deadly form of skin cancer. However, advances in the understanding of its biology have led to the development of several new therapeutic approaches. One of these novel treatment strategies is based on the targeting of the mitochondrial bioenergetic and networks responsible for tumor initiation and progression. Indeed, it has recently emerged that changes in mitochondrial metabolism, dynamics, redox homeostasis, and apoptosis are strictly associated with tumor growth, metastasis, and drug resistance. In this review, we summarize current evidence about the multiple biological functions exerted by mitochondria in melanoma, also focusing on the role of these organelles as promising targets for pharmacological intervention.

## Introduction

Melanoma is the deadliest form of skin cancer [1]. Although it can be effectively treated with surgical removal when detected early at the primary site, its prognosis significantly worsens at advanced stages [2]. Current treatments for metastatic melanoma—such as BRAF and mitogen-activated protein kinase kinase (MEK) inhibitors, as well as immune checkpoint blockade—show inconsistent response rates, and most patients eventually develop resistance to these therapies [2]. This highlights an urgent need to uncover the biological mechanisms driving melanoma aggressive behavior. In this regard, recent research has been focused on the study of the metabolic features of melanoma cells. In particular, alterations in mitochondrial bioenergetics and networks have been linked to cancer metastasis and drug resistance [3, 4]. In the present article, we provide an overview of the different roles played by mitochondria in melanoma development and evolution. Specifically, the function of these organelles in determining metabolic plasticity and redox homeostasis is discussed, together with their ability to regulate apoptosis. The molecular bases of mitochondrial dynamics and quality control are also summarized, with particular emphasis on their involvement in the determination of melanoma cell state and fate. Finally, we explore how targeting mitochondria could be exploited for therapeutic benefit.

## Mitochondrial metabolism in melanoma

As extensively reviewed in [5, 6], melanoma exhibits profound metabolic reprogramming involving glycolysis, fatty acid β-oxidation (FAO), and amino acid catabolism through oxidative deamination and transamination. Regardless of the carbon source, intermediates from these pathways feed into the tricarboxylic acid (TCA) cycle, generating substrates for the electron transport chain (ETC) to fuel oxidative phosphorylation (OXPHOS) [3, 4]. Notably, both the TCA cycle and OXPHOS are dysregulated in melanoma.

The TCA cycle, located in the mitochondrial matrix, serves as a central hub for catabolic and anabolic processes [7]. Among its enzymes, isocitrate dehydrogenase 2 (IDH2) has emerged as a key player in melanomagenesis. Indeed, recent studies have identified sporadic mutations in *IDH* genes in melanoma, suggesting they may confer a growth advantage, mainly in BRAF-mutant tumors [8–10]. In particular, IDH2 and the cytosolic isozyme IDH1 appear to be relevant under hypoxic conditions, where they drive a reversal of TCA flux through reductive carboxylation, shifting lipid biosynthesis from glucose to glutamine dependence [11–13]. Similarly, other enzymes regulating the TCA cycle, such as pyruvate dehydrogenase subunit 1 (PDHA1) and oxoglutarate dehydrogenase (OGDH), are found to be hyperactivated in melanoma, highlighting the importance of citrate metabolism in shaping the tumor metabolic landscape [14]. Despite these observations, recent research has shown that high amounts of citric acid inhibit the growth of different tumor types, mainly by disrupting ATP production, inactivating the insulin-like growth factor 1 receptor (IGF-1R)/Akt pathway and stimulating cell death via downregulation of the anti-apoptotic proteins B-cell lymphoma-extra large (Bcl-xL) and myeloid cell leukemia-1 (Mcl-1) [15]; in melanoma, elevated doses of this metabolite have been demonstrated to suppress cell proliferation and promote UV-induced apoptosis by blockade of the glycogen synthase kinase-3 beta (GSK3β)/β-catenin pathway [16, 17]. Overall, these seemingly contradictory effects suggest a dose-dependent role for citrate, making it a potential therapeutic target and/or agent in melanoma.

**Fig. 1:**
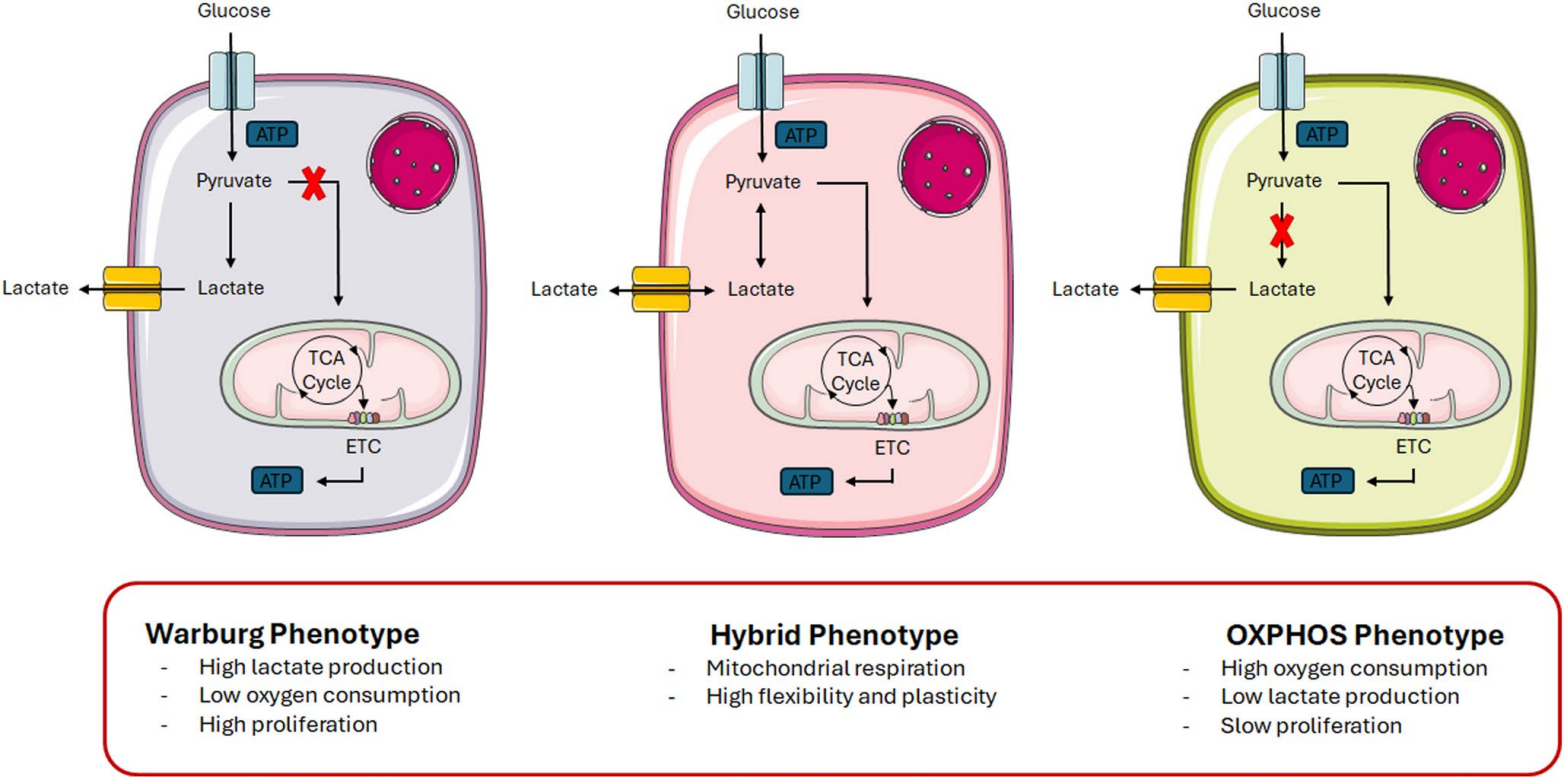
Melanoma is characterized by metabolic plasticity. In 1956, Otto Warburg reported that, in contrast to non-malignant cells, cancer cells display a glycolytic phenotype characterized by high lactate production and low oxygen consumption even in normoxic conditions. However, in the last years it has clearly emerged that melanoma cells have a hybrid phenotype, in which mitochondrial respiration is still functional, allowing high flexibility and plasticity

Mitochondria are the primary cellular consumers of oxygen, which is mainly utilized in adenosine triphosphate (ATP) production via OXPHOS [18]. Under hypoxic conditions, cells switch from OXPHOS to glycolysis to maintain energy homeostasis [18]. In 1956, Otto Warburg described a hallmark of cancer metabolism: the tendency of tumor cells to favor glycolysis over OXPHOS even in the presence of oxygen—a phenomenon known as “aerobic glycolysis” or the “Warburg effect” [18]. Like many cancers, melanoma demonstrates this metabolic phenotype [11, 19–21]. In particular, in melanoma cells aerobic glycolysis is driven by oncogenic activation and loss of tumor suppressors. For example, BRAF mutations, present in nearly 60% of melanomas, enhance glycolytic flux via stabilization of hypoxia-inducible factor 1α (HIF1α) and simultaneous inhibition of mitochondrial respiration through suppression of the microphthalmia-associated transcription factor (MITF)/peroxisome proliferator-activated receptor-γ coactivator 1α (PGC-1α) pathway [22, 23]. Similarly, mutations in phosphate and tensin homolog (PTEN)—which occur in 40–60% of melanomas—lead to persistent Akt activation, upregulating glucose uptake and consumption through increased expression of glucose transporter type 1 (GLUT1), hexokinase 2 (HK2), phosphofructokinase 1 (PFK-1) and lactate dehydrogenase A (LDH-A) [24]. However, growing evidence suggests that OXPHOS remains active and even upregulated during melanoma progression, despite the Warburg effect [25, 26]. Rather than adhering strictly to one metabolic strategy, melanoma cells display a hybrid phenotype that allows flexibility in response to fluctuating nutrient and oxygen availability. When glucose is scarce, cells rely more on mitochondrial respiration; under hypoxia, they shift toward glycolysis, resulting in uncoupled OXPHOS activity [11]. This metabolic heterogeneity is spatially reflected within tumors: cells in poorly vascularized cores primarily depend on glycolysis, while those at better-perfused peripheries favor OXPHOS [26]. Intriguingly, these distinct cellular populations may cooperate metabolically: glycolysis-derived lactate from hypoxic cells can be taken up by neighboring cells to fuel their mitochondria [26]. Adding further complexity, melanoma-associated fibroblasts (MAFs) also contribute to tumor metabolism by entering a catabolic state and secreting metabolites that support the anabolic needs of cancer cells [27–29]. This dynamic interplay of metabolic pathways—termed “metabolic plasticity”—is illustrated in Fig. 1.

As noted earlier, melanoma cells exhibit a remarkable ability to reprogram their metabolism in response to a wide range of both intracellular and extracellular stimuli. In this scenario, both glutaminolysis and FAO play a pivotal role in sustaining the activity of the TCA cycle and OXPHOS. Focusing on the first, melanoma cells often exhibit a strong dependency on glutamine, a phenomenon commonly referred to as "glutamine addiction". This is largely driven by cataplerotic reactions involving glutamate and asparagine, which serve to replenish TCA cycle intermediates while also supporting the synthesis of nucleotides, amino acids, and other macromolecules [30]. Notably, this reliance on glutamine metabolism becomes even more pronounced during the development of resistance to targeted therapies, indicating a metabolic shift that supports cell survival upon drug-induced stress [31]. Turning to FAO, this pathway similarly contributes to melanoma cell proliferation, particularly under conditions of glucose deprivation; in particular, it provides an alternative energy source that helps maintain ATP production and redox balance [32]. Beyond its role in energy metabolism, FAO has also been implicated in enhancing the migratory capacity of melanoma cells, especially those that are resistant to anoikis, a form of programmed cell death triggered by detachment from the extracellular matrix [33, 34]. Moreover, FAO modulates the response of BRAF-mutant melanoma cells to MAPK pathway inhibitors, thereby contributing to therapeutic resistance and disease progression [35]. Taken together, this evidence points to a dual dependency in melanoma metabolism, where both glutamine and fatty acid catabolism is crucial for energy production and survival under stress. Clinically, this opens the door to combination strategies (*i.e.* BRAF or MAPK inhibitors with metabolic inhibitors targeting glutaminase or FAO enzymes) that may prevent or delay drug resistance, improve treatment efficacy, and potentially impair metastatic spread.

Within melanoma microenvironment, metabolic plasticity also orchestrates the interactions between cancer cells and immune components. Specifically, the upregulation of glycolysis in melanoma cells produces a glucose-deprived microenvironment, contributing to the functional exhaustion of CD4 + T lymphocytes [36, 37]. Additionally, the accumulation of lactate is associated with a marked reduction in both the number and activity of cytotoxic CD8 + T cells and natural killer (NK) cells as well as with the polarization of macrophages toward an immunosuppressive, pro-tumoral M2-like phenotype characterized by increased vascular endothelial growth factor (VEGF) expression [38, 39]. However, tumor cells with high OXPHOS levels have also been shown to correlate with resistance to anti-PD-1 immunotherapy; indeed, they seem to promote the development of a hypoxic stroma, culminating in the dedifferentiation and impaired activation of T cells [40]. These immunosuppressive effects highlight how tumor-derived mitochondrial alterations can hinder antitumor immunity. Consequently, strategies aimed at targeting metabolic reprogramming may enhance the effectiveness of immunotherapeutic approaches in melanoma.

## Mitochondrial dynamics and quality control in melanoma

**Fig. 2:**
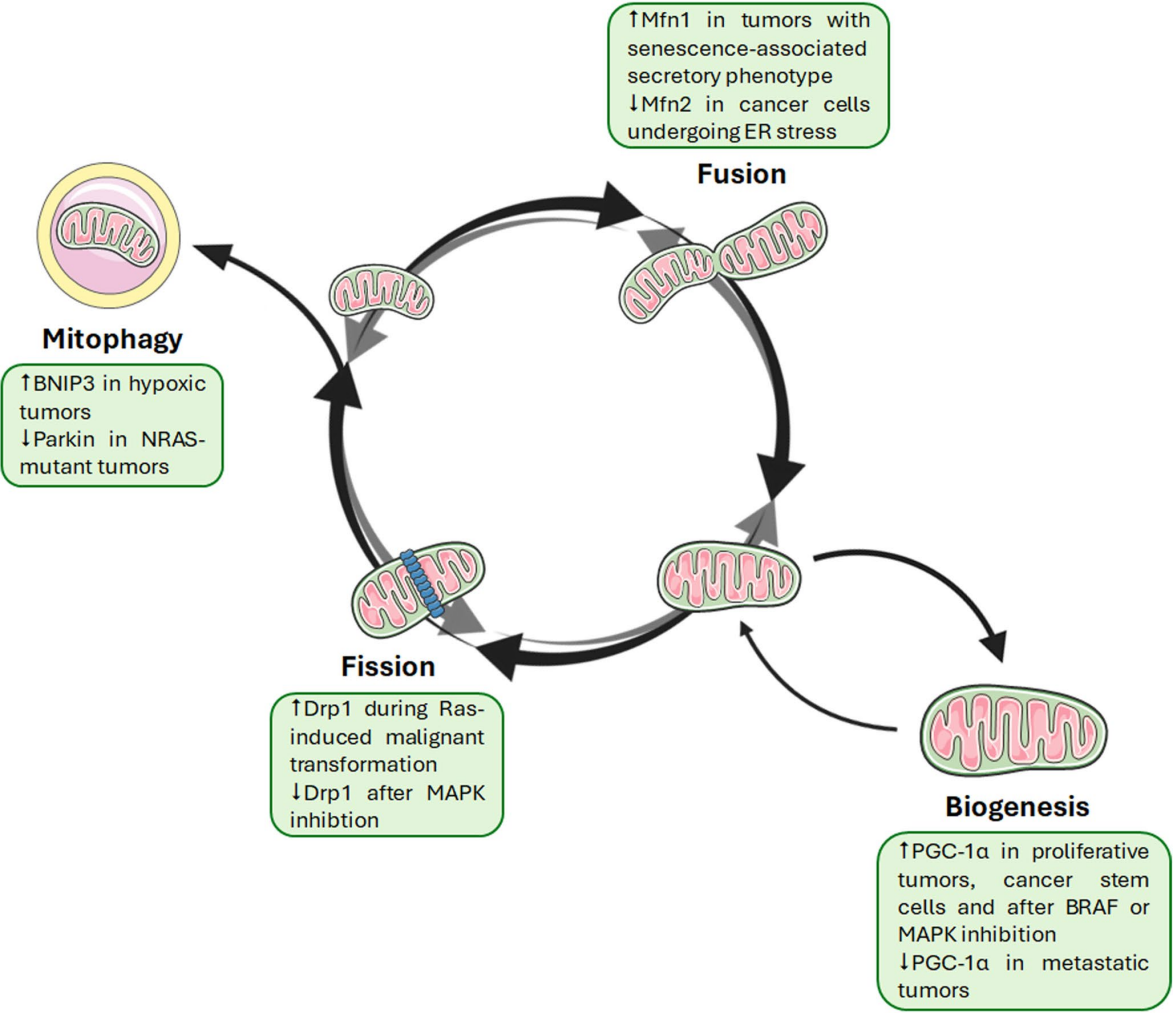
Mitochondrial dynamics are dysregulated during melanoma development and progression. Mitochondria are highly dynamic organelles undergoing continuous processes of biogenesis, fission, fusion, and mitophagy. In melanoma, PGC-1α-driven mitochondrial biogenesis is activated in proliferative tumors, cancer stem cells, and after BRAF or MAPK pharmacological inhibition, while being suppressed during metastasis. Drp1, the main regulator of mitochondrial fission, is upregulated upon Ras-induced malignant transformation, while being inactivated by MAPK inhibitors. Mfn1-related mitochondrial fusion correlates with a senescence-associated secretory phenotype; on the other hand, Mfn2 is degraded in cancer cells undergoing ER stress, leading to mitophagy. Regarding the latter, it seems to be non-canonical, with Parkin being absent in NRAS-mutant malignancies and BNIP3 being overexpressed in hypoxic conditions

Mitochondrial dynamics encompass biogenesis, fission, fusion, and mitophagy—processes essential for maintaining mitochondrial quality control and overall cellular health [30]. These mechanisms play critical roles in melanoma initiation and progression (Fig. 2).

PGC-1α is a key transcriptional coactivator regulating mitochondrial biogenesis [41]. Upon nuclear translocation, it activates nuclear respiratory factors (Nrf1 and Nrf2) and mitochondrial transcription factor A (TFAM), promoting mitochondrial protein synthesis and organelle proliferation. PGC-1α also interacts with various nuclear receptors—peroxisome proliferator-activated receptors (PPARs), estrogen-related receptors α (ERRs), hepatic nuclear factors (HNFs), liver X receptors (LXRs), farnesoid X receptor (FXR), retinoic acid receptor (RARα), and the glucocorticoid receptor—to influence cellular energy metabolism beyond the mitochondria [42]. Remarkably, Vazquez et al. have recently reported that PGC-1α is overexpressed in a subset of melanomas displaying MITF activation, where it correlates with improved tolerance to the damaging effects of reactive oxygen species (ROS) and increased ability to survive under conditions of oxidative stress [43]. Intriguingly, PGC-1α expression also displays high intratumor heterogeneity, with cellular populations with low levels of this protein displaying a pro-metastatic gene program and subgroups with high PGC-1α activity driving a proliferating phenotype [44–46]. In line with these reports, this transcriptional coactivator has been found to be upregulated in the melanoma stem cell niche, highlighting the cruciality of mitochondrial content in determining tumor relapse [47]. Finally, increased MITF/PGC-1α-driven OXPHOS has been observed after treatment with BRAF and mitogen-activated protein kinase (MAPK) inhibitors [23, 48–50]. Collectively, these observations highlight the ability of mitochondrial biogenesis to redirect melanoma metabolic phenotypes and confer a significant survival advantage to cancer cells; thus, understanding the context-dependent role of this process should be considered of primary importance in developing effective targeted therapies.

Mitochondrial shape, tightly controlled by the balance of fission and fusion, reflects and regulates metabolic activity and apoptosis [51]. Fission is mediated by Drp1, which relocates to the mitochondrial outer membrane (MOM) and interacts with fission-1 (Fis1) and mitochondrial fission factor (Mff). Fusion involves mitofusin 1/2 (Mfn1/2) on the MOM and optic atrophy 1 (OPA1) on the inner membrane [51]. Notably, mitochondrial morphology transitions regulate melanin synthesis via ROS generation in melanocytes [52, 53]. In melanoma, Drp1-dependent mitochondrial division is required for RAS-induced malignant transformation [54]. In addition, it is inhibited by MAPK pathway inhibitors, leading to mitochondrial superfusion and enhanced oxidative capacity [55, 56]. On the other hand, Mfn1 knockdown suppresses melanoma senescence-associated secretory phenotype (SASP), delaying tumor growth after chemotherapy and promoting immune cell recruitment [57]. In light of these considerations, any disruption of the balance between fusion and fission can profoundly affect the development, progression, and treatment of melanoma. Therefore, the design of drugs selectively targeting mitochondrial shape represents a promising avenue for melanoma therapy; as discussed later in this manuscript, this can be realized either by re-establishing fusion/fission homeostasis to block cell proliferation, migration, and drug resistance, or by altering fusion/fission balance to induce apoptosis.

Mitophagy, a selective form of autophagy, clears damaged mitochondria to maintain cellular homeostasis [58]. The PTEN-induced kinase 1 (PINK1)/parkin pathway is the best-characterized mitophagic mechanism: under stress, PINK1 accumulates on mitochondrial surface, recruits and phosphorylates parkin, and triggers ubiquitination of MOM proteins, leading to mitophagosome formation via the microtubule-associated protein light chain 3 (LC3)-mediated interactions with adaptor proteins, such as p62 [58]. Of note, parkin protein is expressed in melanocytes but not in melanoma cells, and its expression decreases upon melanocyte transformation by NRAS mutation; downregulation of parkin in melanocytes stimulates their proliferation, while its re-expression in melanoma leads to cell cycle arrest [59]. This has raised the hypothesis that parkin might function as tumor suppressor in melanoma. Furthermore, it seems to suggest that in this tumor the mitophagic flux might be PINK1/parkin-independent. In this regard, Vara‐Pérez and colleagues have demonstrated that Bcl-2-interacting protein 3 (BNIP3), which can recruit LC3 irrespective of PINK1/parkin cascade initiation, is activated in response to hypoxia [60]; this results in mitophagy induction, which alleviates ROS-associated mitochondrial dysfunction and enhances OXPHOS, while parallelly inhibiting glycolysis via impairment of HIF-1α stability [60, 61]. Likewise, Wang and coworkers have shown that, under endoplasmic reticulum (ER) stress, the unfolded protein response (UPR) induces the transcription of mitochondrial E3 ubiquitin-protein ligase (MARCH5) to promote the ubiquitination and degradation of Mfn2, resulting in the activation of non-canonical pro-survival mitophagy [62]. Despite still scanty, these preliminary studies highlight that mitochondrial biogenesis, dynamics, and mitophagy are all implicated in melanoma onset and evolution. Importantly, the three mitochondrial processes are interrelated, indicating that their dynamic interplay might hide great potential as therapeutic target. Based on these premises, further research is encouraged to dissect the complex function of mitophagy in melanomagenesis.

As discussed above, mitochondrial integrity is preserved via mitochondrial biogenesis, activated in response to context-specific cellular demands, and mitophagy, triggered by mitochondrial dysfunction [41]. If mitochondrial damage prevention and repair fail, cells can exchange copies of mtDNA, which encodes the peptides essential for OXPHOS [63, 64]. Intriguingly, mitochondrial transfer from the endothelium to melanoma has been found to promote cancer cell proliferation via Nrf2/HO-1 pathway and to induce M2 macrophage polarization [65]. Moreover, human melanoma cells can attract bone marrow-derived mesenchymal stem cells (MSCs) to the primary tumor site, stimulating mitochondrial biogenesis in the latter through PGC-1α upregulation; mitochondria are then taken up by tumor cells via direct contact with MSCs [66]. Likewise, MSCs in mouse melanoma microenvironment can directly transmit mtDNA to mitochondria-deprived cancer cells, conferring respiration rates similar to those of their parental counterpart [67]. Of course, further studies are required to validate the role of mitochondrial trafficking in the clinical setting.

Mitochondria-derived vesicles (MDVs) are a type of extracellular vesicles (EVs) that originate from mitochondria. They are formed from mitochondrial sub-compartments and released into the cytosol, and can also be incorporated into larger EVs and excreted from the cell. MDVs are deeply implicated in mitochondrial quality control, intercellular communication, and potentially in various diseases. In this regard, it should be noted that functional mitochondria are actively transported by melanoma EVs and lipidosomes, influencing both donor and recipient cells and significantly impacting tumor growth and dissemination [68, 69]. Moreover, it has been recently reported that adipocyte-derived EVs containing mitochondrial proteins involved in FAO are transferred to melanoma, thus fueling cancer metastasis; interestingly, this might in part explain why obese melanoma patients have a poorer prognosis than non-obese subjects [70]. Although still preliminary, this evidence suggests that pharmacologically blocking MDV formation or altering EV content might prevent tumor progression. Furthermore, MDV protein signatures could serve as novel diagnostic or prognostic markers for melanoma.

It is now well-established that some malignancies, including thyroid, renal, and colorectal carcinoma, accumulate loss-of-function mtDNA mutations and adopt an oncocytic phenotype characterized by high mitochondrial content [71]. In contrast, such mutations have been found to suppress metastasis and enhance immunotherapy response in melanoma, suggesting that functional mitochondria are required for the progression of this tumor [72, 73]. In this setting, analysis of mtDNA heteroplasmy, commonly observed in inherited mitochondrial diseases, could offer interesting insights into tumor selection for functional mitochondrial genomes [74]. Remarkably, advanced multi-omics techniques, such as DOGMA-seq and PHAGE-ATAC, enable simultaneous profiling of mtDNA, RNA, and protein content at single-cell resolution, facilitating exploration of mtDNA influence on melanoma heterogeneity and treatment outcomes [75].

Mitochondria contain 13 proteins encoded by mtDNA and more than 1000 proteins encoded by nuclear DNA [76]. Within the mitochondrial matrix, ATP-dependent proteases, such as mitochondrial Lon peptidase 1 (LONP1) and caseinolytic protease X and protease P complex (CLPXP), maintain mitochondrial homeostasis by eliminating damaged or misfolded proteins [76, 77]. Interestingly, clinical studies indicate that LONP1 overexpression is a poor prognosis marker in melanoma; in particular, functional experiments have revealed that it promotes tumor growth and metastasis and protects against senescence [78]. On the other hand, high levels of ClpP correlate with a shorter survival of patients with uveal melanoma [79]. Although the molecular mechanisms underlying the pro-tumor activity of mitochondrial proteases still need to be clarified, the above findings not only demonstrate the relevance of these molecules for cellular and organismal viability but also identify them as crucial regulators of melanomagenesis.

As shown in the previous paragraphs of this chapter, mitochondria dynamically adapt to the changing environment by adjusting their nucleic acid and protein contents. Additionally, the mitochondrial components are modulated in terms of activity and interactions. In this context, multi-omics approaches offer comprehensive insight into mitochondrial regulation in melanoma [75]. Genomics identifies mutations in both mtDNA and nuclear-encoded mitochondrial genes; transcriptomics tracks mitochondrial gene expression changes, while ribosome profiling and mePROD assess mitochondrial protein synthesis and import; PTM profiling reveals regulatory modifications, complexome profiling maps protein supercomplexes, and N-terminomics measures mitochondrial protease activity [75]. The major advantage of these techniques is that they can examine thousands of cellular macromolecules at once. Thus, multi-omics approaches not only offer a more holistic view of melanoma mitochondrial networks but also hold promise for diagnostic applications by linking metabolic phenotypes to molecular signatures.

## Mitochondrial redox homeostasis in melanoma

**Fig. 3:**
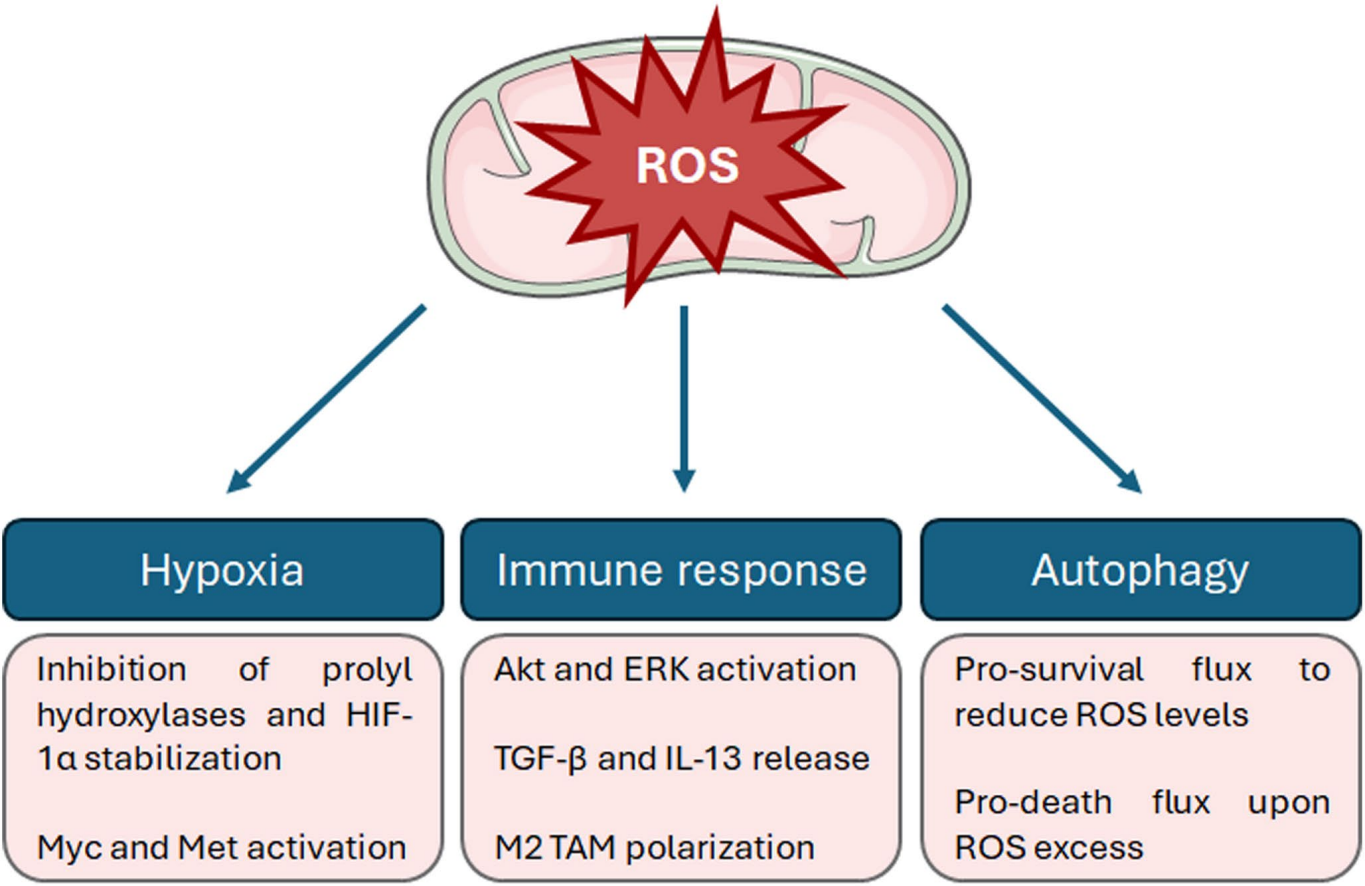
Mitochondrial ROS regulate several tumorigenic processes in melanoma. Mitochondrial ROS have been found to exert a pleiotropic role in melanoma. First, they inhibit prolyl hydroxylases, leading to HIF-1α stabilization and contributing to Myc and Met activation. Then, they activate the Akt and ERK pathways, resulting in TGF-β and IL-13 release and subsequent M2 TAM polarization. Finally, they control the autophagic flux, determining cell survival or death

ROS are widely recognized as key drivers of melanomagenesis, due to their ability to damage DNA and induce genetic and epigenetic alterations associated with cancer development [80]. Beyond genomic instability, oxidative stress promotes metabolic reprogramming—particularly enhancing glucose metabolism—and contributes to immunosuppression, thereby accelerating melanoma progression [80]. While the exact sources of ROS overproduction in melanoma remain unclear, UV radiation, melanin biosynthesis, and NADPH oxidase (NOX) activity have been identified as primary contributors to redox imbalance [80, 81]. Importantly, mitochondria also play a significant role in ROS generation in melanoma cells (Fig. 3).

The relationship between mitochondrial ROS and HIF-1α has been largely investigated in melanoma. In particular, mitochondrial oxidative stress has been found to stabilize HIF-1α protein by inhibiting prolyl hydroxylases [82]. This interplay has been confirmed in a Myc-dependent mouse model of melanoma, where different antioxidants, including N-acetylcysteine and vitamin C, could successfully suppress tumor growth [83]. Other studies have shown that ROS-mediated stabilization of HIF-1α can lead to activation of the proto-oncogene Met, which promotes the aggressiveness of early-stage tumors, partially through vasculogenic mimicry [84]. Of note, HIF-1α upregulation correlates with melanoma poor prognosis, contributing to cancer progression not only by inducing a variety of other pro-tumor pathways, such as the RAS/RAF/MEK/extracellular signal-regulated kinase (ERK), phosphatidylinositol 3-kinase (PI3K)/Akt/mechanistic target of rapamycin (mTOR), Janus kinase (JAK)/signal transducer and activator of transcription (STAT), Wnt/β-catenin, Notch and nuclear factor kappa-light-chain-enhancer of activated B cells (NF-κB) cascade, but also by regulating the levels of different oncogenic miRNAs [85]; whether ROS from mitochondria are involved in the control of this intricate molecular network remains to be elucidated.

In addition to modulating transcriptional programs, mitochondrial ROS facilitate the release of inflammatory cytokines, such as transforming growth factor beta (TGF-β) and interleukin 13 (IL-13), from melanoma cells. In particular, both the ERK and Akt pathways appear to positively regulate the induction of this inflammatory state [86]. This results in the activation and M2 polarization of tumor-associated macrophages (TAMs), determining the establishment of an immunosuppressive microenvironment that facilitates cancer growth and metastasis [65]. These findings support the dual role of ROS in cancer: while acute exposure can stimulate innate and adaptive immune responses, chronic ROS generation may lead to T-cell exhaustion and reduced responsiveness to immunotherapy [87]. Thereby, carefully modulating oxidative stress could boost anti-tumor immunity and improve the efficacy of current melanoma treatments.

ROS also play a critical role in regulating cellular stress responses, particularly through the activation of autophagy—a major pathway for maintaining cellular homeostasis [88]. In melanoma, mitochondrial ROS generated during hypoxia or ER stress can induce pro-survival autophagy, facilitating the rapid clearance of damaged components through lysosomal degradation [89, 90]. However, excessive or sustained autophagy can shift from a protective mechanism to a cytotoxic one, leading to cell death due to over-degradation of cellular structures [89, 90]. As explored later in this review, emerging anti-melanoma therapies are increasingly focused on targeting autophagy as a means of controlling tumor growth.

## Mitochondrial apoptosis in melanoma

**Fig. 4:**
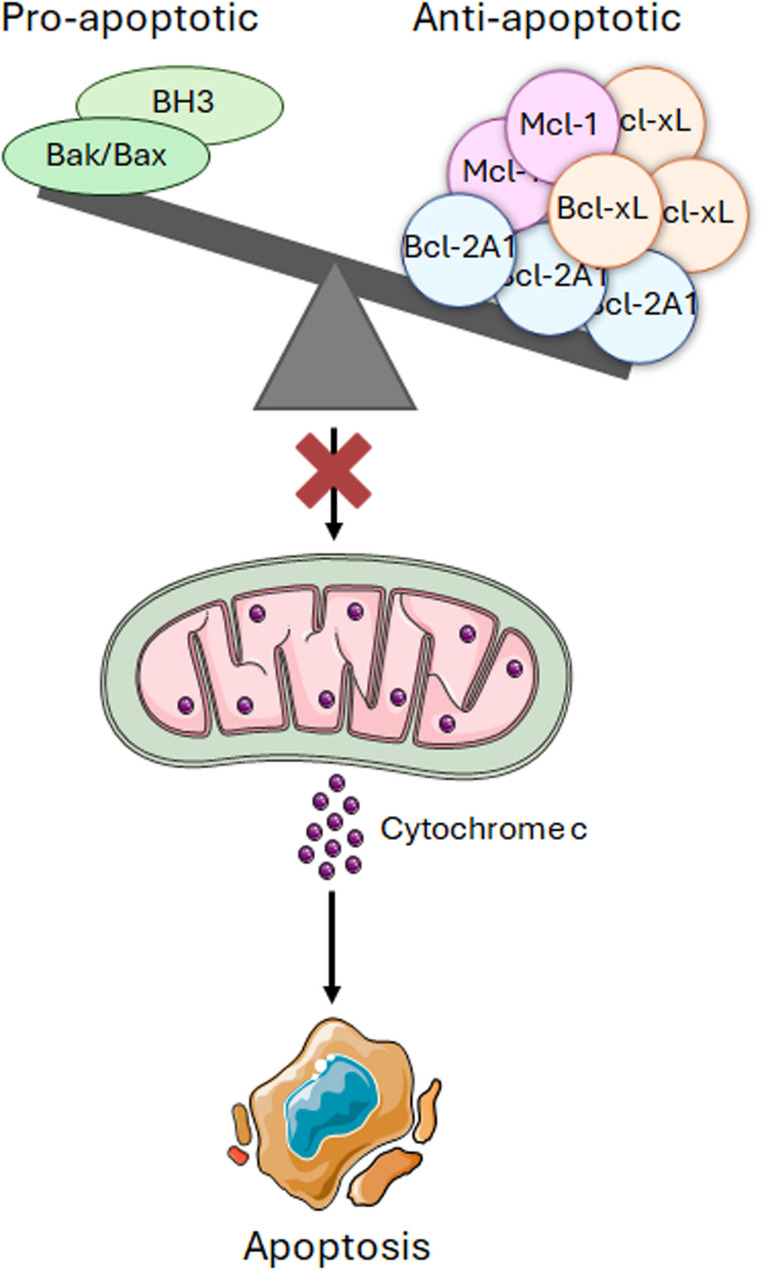
Mitochondrial apoptosis is reduced in melanoma. Melanoma is characterized by the upregulation of different anti-apoptotic proteins belonging to the Bcl-2 mitochondrial family, including Bcl-2A1, Bcl-xL and Mcl-1. Targeting these proteins with ad hoc drugs might result in improved therapeutic outcomes

Apoptosis, or programmed cell death, is a fundamental biological process essential for embryonic development, tissue homeostasis, and the removal of damaged or abnormal cells [91, 92]. There are two primary apoptotic pathways: the extrinsic pathway, initiated by the binding of death ligands to membrane-located death receptors, and the intrinsic (mitochondrial) pathway, triggered by internal stress signals, such as nutrient deprivation, DNA damage, oxidative stress, or infection [91, 92]. The intrinsic pathway is tightly regulated by the Bcl-2 family of proteins, which includes both anti-apoptotic members (Bcl-2A1, Bcl-xL, Mcl-1) and pro-apoptotic effectors (Bcl2-Associated X protein—Bax—and Bcl-2 homologous antagonist/killer—Bak). The balance between these opposing groups determines mitochondrial outer membrane permeabilization (MOMP). When pro-apoptotic signals prevail, cytochrome c is released from mitochondria into the cytoplasm, where it associates with apoptotic protease-activating factor-1 (APAF-1) to form the apoptosome, leading to caspase-9 activation and subsequent execution of apoptosis [91–93]. In melanoma, several members of the Bcl-2 family are dysregulated, contributing to therapy resistance and disease progression. For example, Bcl-2 is more highly expressed in melanocytes than in many other cell types, and its relative *BCL2A1* is amplified in 30–40% of melanomas. This amplification enhances resistance to cytotoxic chemotherapy and targeted agents [94–96]. Similarly, Bcl-xL is frequently overexpressed in metastatic melanoma and is associated not only with resistance to apoptosis and drug treatment but also with cancer cell stemness, angiogenesis, and immune evasion; indeed, it has been recently reported that it promotes melanoma cell phenotypic plasticity as well as the release of different pro-angiogenic and inflammatory factors from cancer cells, including VEGF, IL-1β e IL-8 [97–103]. Another key anti-apoptotic protein, Mcl-1, plays a particularly important role in BRAF-mutant melanomas; in these tumors, MAPK pathway hyperactivation drives both increased *MCL1* transcription and stabilization of Mcl-1 protein through phosphorylation at threonine 163, preventing its degradation [104–108]. As illustrated in Fig. 4, the central role of Bcl-2 family proteins in regulating cell death and survival in melanoma makes them promising targets for novel therapies. In particular, inhibitors of Bcl-2, Bcl-xL, or Mcl-1 may help to overcome drug resistance in tumors that have relapsed following conventional treatment.

## Mitochondria in melanoma therapy

Based on the above evidence, many new anticancer therapies are currently targeting mitochondria in the attempt to eradicate melanoma (Table 1).

**Table 1:** Mitochondrial inhibitors in melanoma therapy

Drug	Mechanism of action	Clinical development	References
CPI613	PDHA1 and OGDH inhibition	Preclinical studies	[14]
BAY 87-2243, IACS-010759, metformin, and phenformin	OXPHOS inhibition	Preclinical studies; phase I/II trial (metformin)	[109–120]
Mensacarcin, cryptolepine, deguelin, tocotrienols, and limonoids	OXPHOS inhibition	Preclinical studies	[122–126]
Mdivi-1	Drp1 inhibition	Preclinical studies	[127]
ONC201, ONC206, and ONC212	ClpP agonism	Preclinical studies	[130–132]
Tigecycline, doxycycline, and azithromycin	Mitochondrial translation inhibition	Preclinical studies	[131, 133–138]
Elesclomol, simvastatin, resveratrol, curcumin, tocotrienols, and chalcone derivatives	ROS induction	Preclinical studies	[141–145]
SAR405, 3-MA, and chloroquine	Pro-survival autophagy inhibition	Preclinical studies	[147–149]
Everolimus, GSK621, and trehalose	Pro-death autophagy induction	Preclinical studies; phase II trial (Everolimus)	[150–153]
Antisense oligonucleotides	Bcl-2 and Bcl-xL inhibition	Preclinical studies	[154, 155]
A-1331852, GX15-070 (Obatoclax), ABT-263 (Navitoclax), and ABT-737	BH3 mimicry	Preclinical studies; phase I/II trial (Navitoclax)	[156–159, 162]
SC-2001, S63845 and S64315 (MIK655)	Mcl-1 inhibition	Preclinical studies	[160, 161]
Paraptosis inducers	Ca^2+^ overload-dependent mitochondrial swelling	Preclinical studies	[163]
Ferroptosis inducers	Intracellular iron accumulation and lipid peroxidation	Preclinical studies	[164]

Both TCA and OXPHOS have been exploited for melanoma treatment. In particular, CPI613, a lipoic acid analog that inhibits PDHA1 and OGDH, has been shown to dramatically attenuate the progression of melanoma and improve the therapeutic efficacy of anti-PD-1 against this tumor [14]. On the other hand, several inhibitors of the OXPHOS complex I, such as BAY 87–2243, IACS-010759, metformin and phenformin, have been observed to be potent melanoma suppressors [109–113]. Of note, these chemicals also display strong synergism when administered in combination with both targeted and immunotherapies, due to their ability to specifically target the metabolic signatures associated with BRAF and MAPK inhibitor resistance on the one side, and to alleviate the hypoxia responsible for CD8 + T cell dysfunction of the other side [110, 114–120] (ClinicalTrials.gov, NCT04114136, NCT03311308). However, their use remains limited because of their high toxicity; indeed, due their broad and unspecific activity, they commonly cause fatigue, nausea, vomiting, diarrhea, lactic acidosis, elevated blood lactate, and peripheral neuropathy [121]. In this regard, it should be noted that several mitochondria-targeting natural compounds free of side effects are now under study for melanoma therapy; among them, mensacarcin, cryptolepine, deguelin, tocotrienols, and limonoids have demonstrated great anticancer potential [122–126].

Drp1 has been recently proposed as a promising target for melanoma management. Indeed, Akita et al. have found that mitochondrial division inhibitor-1 (mdivi-1), a specific Drp1 inactivator, sensitizes melanoma cells to apoptosis induced by the tumor necrosis factor-related apoptosis-inducing ligand (TRAIL), by causing robust mitochondrial hyperfusion and eliciting both the ER and oxidative stress responses. Importantly, mdivi-1 cytotoxic effects are minimal in normal melanocytes and fibroblasts, suggesting the possibility to exploit melanoma cell susceptibility to mitochondrial breakdown to improve therapy outcomes [127].

Mitochondrial UPR (mtUPR) occurs when an excess of unfolded or misfolded proteins accumulates in the mitochondrial matrix, eventually culminating into mitochondrial dysfunction [128]. Activation of this pathway by imipridones, a class of ClpP agonists, leads to the eradication of different tumor types, including both solid (*i.e.* glioblastoma and breast, endometrial and prostate carcinoma) and hematological (*i.e.* multiple myeloma and acute myeloid leukemia) malignancies [129]. In melanoma, ONC201, ONC206, and ONC212 displayed potent anti-tumor effects in vivo, a broad therapeutic window and a favorable pharmacokinetic profile [130–132]. Based on these findings, these chemicals warrant further development as anti-melanoma drug candidates.

Inhibition of mitochondrial translation is rapidly emerging as a novel anti-tumor strategy. In melanoma, several antibiotics, such as tigecycline, doxycycline, and azithromycin, have been shown to suppress OXPHOS capacity by reducing the levels of ETC complexes [131, 133–137]. Remarkably, these molecules could selectively eradicate the cancer stem cell niche, known to greatly rely on oxidative metabolism for its own maintenance and propagation [138]. However, it should be noted the antibiotic exposure appears to be associated with an increased incidence of cancer; as a direct consequence of antibiotic misuse, it is highly likely that microbiota dysfunction may enhance cancer risk and limit the efficacy of chemotherapy, radiotherapy, and immunotherapy [139, 140]. In this setting, further studies are required to elucidate the correlation between antibiotic usage and melanoma development.

Given their crucial role in determining cancer cell survival or death, ROS levels can be pharmacologically modulated for melanoma eradication [80]. While the anti-tumor benefits of antioxidants still need to be elucidated, several reports have pointed out that ROS inducers can successfully suppress melanoma growth. Among these molecules, we can count several synthetic drugs, particularly elesclomol and simvastatin, and nutraceuticals, including resveratrol, curcumin, tocotrienols, and chalcone derivatives [141–145]. These chemicals mainly act through ROS-mediated DNA damage, cell cycle arrest, apoptosis, Akt inactivation, and induction of a senescent phenotype [80, 141–145]. Notably, increased ROS-induced melanoma cell death has also been reported after ATP depletion caused by the inactivation of glycolytic enzymes as well as by chemotherapy, radiation therapy, and immunotherapy [80]; these observations highlight the great potential of ROS manipulation in anticancer combinatorial treatments. Although more efforts are needed to enhance the selectivity of anticancer ROS-related drugs, the main mechanisms involved in melanoma cellular stress responses are continuously being unveiled and most of them concern the redox balance.

As previously illustrated, autophagy is a critical process in melanoma, involving autophagosome formation and its subsequent fusion with lysosomes for cargo degradation; from a molecular point of view, its regulation is based on mTOR-dependent and independent pathways, such as adenosine monophosphate-activated protein kinase (AMPK), MAPK, ER, and oxidative stress [90, 146]. In this setting, pro-survival autophagy inhibitors, able to block autophagosome or autolysosome generation, and pro-death autophagy inducers, modulating mTOR-related and unrelated autophagic mechanisms, have proved to be useful against melanoma [90, 146]. Well-known members of the first pharmacological class are SAR405, 3-methyladenine (3-MA), and chloroquine, while Everolimus, GSK621, and trehalose belong to the second one [147–153]. However, clinical translation remains challenging due to the non-specific nature of these agents and the involvement of autophagic proteins in various biological processes [90]. In this context, future therapies may focus on the transient modulation of autophagy rather than on its complete and irreversible blockage or induction. Whether autophagy should be up or downregulated in melanoma is still under debate, particularly given its metabolic state-dependent effects on cancer cells.

Because of its relevance in the evasion of apoptosis, different strategies have been evaluated to inhibit the Bcl-2 protein family in melanoma. These include antisense oligonucleotides targeting Bcl-2 and Bcl-xL mRNAs and small molecules, such as the BH3 mimetics A-1331852, GX15-070 (Obatoclax), ABT-263 (Navitoclax), and ABT-737 [154–159]. Importantly, combinations of BH3 mimetics with Mcl-1 inhibitors, namely SC-2001, S63845, and S64315 (MIK655), are even more effective at eradicating melanoma in vitro and in vivo [160, 161]. Furthermore, combining these drugs with either BRAF or MEK inhibitors resensitizes melanoma cells to the latter and enhances xenograft tumor growth inhibition [162]. In this regard, a phase I/II trial study using Navitoclax in combination with dabrafenib and trametinib is recruiting unresectable or metastatic patients with BRAF mutant melanoma (ClinicalTrials.gov, NCT01989585). Alternative treatment options are based on the induction of non-apoptotic cell death mechanisms, including paraptosis, characterized by calcium (Ca^2+^) overload-dependent mitochondrial swelling, and ferroptosis, caused by intracellular iron accumulation and lipid peroxidation; nonetheless, they have only been tested in preclinical models till now [163, 164].

While promising, mitochondria-targeted therapies still face challenges in selectivity, often leading to side effects in healthy tissues. Nanotechnology presents an innovative solution to this issue, by enhancing drug delivery precision, reducing off-target effects, and improving mitochondrial targeting [165]. Nanocarriers, such as liposomes and polymeric/inorganic nanoparticles, have been designed to cross cell membranes and deliver therapeutic agents directly to mitochondria; the modification of these vectors—in terms of structure, charge, and size—facilitates their accumulation within the mitochondrial matrix [165]. This approach, powered by advancements in materials science, bioengineering, and molecular biology, holds the promise of unlocking new frontiers in melanoma treatment.

## Conclusion and future perspectives

In recent years, advancements in melanoma research have underscored the pivotal role of TCA cycle and OXPHOS in regulating tumor initiation and progression, shifting the focus towards mitochondrial metabolism. This progress has deepened our understanding of mitochondrial dynamics and quality control in the metabolic adaptability of melanoma. Studies have also explored the mitochondrial alterations responsible for redox imbalance and the evasion of apoptosis, revealing new pharmacological targets for treating melanoma. In fact, it has become clear that targeting mitochondrial function could be a highly effective approach for melanoma therapy. Several compounds directed against mitochondrial proteins have shown promise in preclinical studies, although translating these findings into clinical applications remains challenging; currently, only a few of these agents are in early-stage clinical trials. The use of next-generation technologies, such as metabolic profiling and single-cell sequencing, will likely provide further insights into the mechanisms controlling mitochondrial metabolism in melanoma. These technologies could enable more personalized and less toxic treatment options, while combining mitochondrial inhibitors with standard targeted and immunotherapeutic therapies could offer a novel strategy for melanoma management.

## Data Availability

No datasets were generated or analysed during the current study.
